# Research on Food Preservation Based on Antibacterial Technology: Progress and Future Prospects

**DOI:** 10.3390/molecules29143318

**Published:** 2024-07-15

**Authors:** Zejing Chu, Hongsu Wang, Biao Dong

**Affiliations:** 1 College of Food Science and Engineering, Jilin University, Changchun 130062, China; zhuzj9921@mails.jlu.edu.cn; 2 College of Electronic Science and Engineering, Jilin University, Changchun 130062, China

**Keywords:** food preservation, photodynamics, ionizing radiation, antimicrobial peptides

## Abstract

The nutrients present in food are not only prone to a series of physicochemical reactions but also provide conditions for the growth and reproduction of foodborne microorganisms. In recent years, many innovative methods from different fields have been introduced into food preservation, which extends the shelf life while maximizing the preservation of the original ingredients and properties of food. In this field, there is a lack of a systematic summary of new technologies emerging. In view of this, we overview the innovative methods applied to the field of food preservation in recent 3 years, focusing on a variety of technological approaches such as antimicrobial photodynamic therapy based on nanotechnology, electromagnetic radiation sterilization based on radiation technology, and antimicrobial peptides based on biomolecules. We also discuss the preservation mechanism and the application of the different methods to specific categories of products. We evaluated their advantages and limitations in the food industry, describing their development prospects. In addition, as microorganisms are the main causes of food spoilage, our review also has reference significance for clinical antibacterial treatment.

## 1. Introduction

With advancing technology and improved quality of life, the demand for effective food preservation methods is rising. Throughout the stages of food processing, storage, transportation, and retail, preservation remains a pivotal area of research due to environmental factors such as spoilage microorganisms and oxygen, physiological processes such as respiration and maturation of organisms, and the effects of various enzymes. The significance of food preservation extends beyond enhancing sensory qualities and nutritional content to impacting public health and economic outcomes at regional and national levels [1]. Innovations in inhibiting harmful bacteria and antioxidative techniques within food preservation not only bolster food safety but also offer valuable insights applicable to clinical medicine and beyond.

Currently, large-scale commercial food preservation technologies can be categorized into three main types: heating sterilization, low-temperature storage, and chemical preservatives [2]. However, each of these methods has notable drawbacks. Heating sterilization, while effective at eliminating foodborne pathogens and deactivating enzymes, is energy-intensive and increases preservation costs. Moreover, it can compromise food quality by potentially generating carcinogens like nitrites, depleting essential nutrients such as vitamins, denaturing proteins, and diminishing sensory appeal. Low-temperature storage methods such as refrigeration and freezing are adept at slowing microbial growth, inhibiting enzymatic activity, and extending food shelf life. Nevertheless, these techniques fall short of achieving complete sterilization and enzyme deactivation and are limited to temporary food storage. Furthermore, refrigerants like Freons used in cooling equipment can pose environmental risks. Chemical preservatives like sodium nitrite and sodium benzoate are widely employed, despite stringent regulatory controls due to concerns over toxicity and residue levels. Inconsistent global regulations also hinder economic progress. Consequently, there is a pressing need for the development of cost-effective, safer, more convenient, and efficacious food preservation technologies.

Considering that food microorganisms are one of the main causes of food spoilage [3], this article will mainly focus on sterilization, discussing several new sterilization methods that destroy bacterial cell membranes, proteins, DNA, and other biomolecules, such as antimicrobial photodynamic therapy (aPDT), ionizing radiation (IR) sterilization, and antimicrobial peptides (AMPs). Based on the destruction of spoilage bacterial biomolecules, this article summarizes relevant research in the field of food preservation in the past five years and predicts the development trend of food preservation in the next few years.

It is noteworthy that traditional packaging methods typically utilize physical or chemical means to enclose food within materials providing functions, such as moisture resistance, oxidation prevention, and preservation, yet often lack antibacterial properties. The incorporation of substances like PS (photosensitizers) and AMPs (antimicrobial peptides) into food packaging enables the implementation of antibacterial technologies such as antimicrobial photodynamic therapy (aPDT). Packaging nanomaterials, whether in the form of films or wraps, offer advantages such as protective capabilities, targeted action, controlled release, and adjustability. Recent research has demonstrated the achievement of highly selective inhibition of specific microorganisms through the formulation of food packaging containing tailored carriers of PS, AMPs, and similar agents, thereby precisely regulating their release dynamics [4].

## 2. Antibacterial and Food Preservation Based on aPDT

aPDT has evolved beyond its origins in clinical medicine, where it was initially used to deactivate pathogens and tumor cells. Today, aPDT has found expanded applications in various sterilization-related fields, including the food industry. Particularly noteworthy is its role in addressing food safety concerns by effectively inactivating pathogenic microorganisms within food products. Vinayak S. Ghate et al. [3] summarized and analyzed the relevant research on aPDT inactivating pathogenic microorganisms in the field of food safety from 2014 to 2019 and proposed that the future development direction of aPDT in the food industry is to continuously explore new photosensitizers (PS) for technological innovation, gradually achieving commercial application. Its cost is very low, for example, the sterilization cost per pig is less than $1. Until the past three years, more research has focused on inactivating spoilage-causing microorganisms using aPDT, which has now begun to emerge in the field of food preservation. For instance, the primary mechanisms through which aPDT effectively combats pathogenic bacteria and spoilage microorganisms on various food surfaces and contact materials include destroying cell structure and function, oxidizing macromolecules, inhibiting quorum sensing, dismantling biofilms, and reducing virulence factors. Additionally, owing to its multi-target approach, aPDT minimizes the likelihood of drug resistance [3]. In addition to these theoretical studies, an increasing number of studies are exploring the application of aPDT in specific foods. Liu Dan et al. [5] summarized and organized the development of photosensitizers and the selection of light sources and introduced the application of aPDT in food preservation from four types of food matrices: vegetables, fruits, seafood, and poultry, especially the different application scenarios of natural photosensitizers such as chitosan. This section will focus on summarizing the application of aPDT in the field of food preservation in the past three years through the types of photosensitizers and food substrates.

### 2.1. Mechanism of aPDT

**Figure 1:**
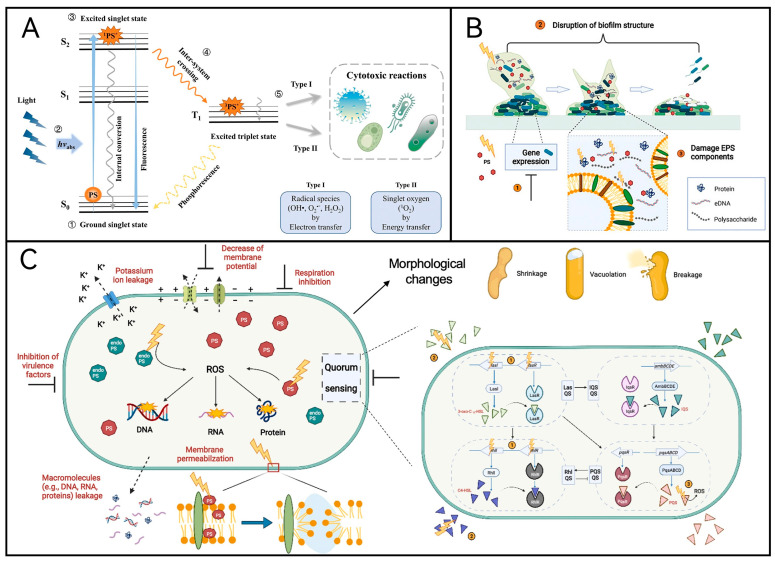
(**A**) The process of action of aPDT [10]. (**B**) aPDT damages biofilms in three ways [11]. (**C**) Antimicrobial mechanisms of aPDT against planktonic cells. Upon the aPDT, three major morphologic have changed: cell shrinkage, formation of vacuoles, and cell breakage/leakage of intracellular contents [11].

In brief, photodynamic inactivation technology operates on the principle that photosensitizers undergo energy-level transitions upon absorbing specific light wavelengths. This process generates reactive oxygen species through either electron transfer or energy transfer mechanisms, thereby oxidizing macromolecules within cells and resulting in their demise (Figure 1A). Upon the aPDT, it damages biofilms through (i) modulation of gene expressions of bacterial cells in the biofilm, (ii) disruption of biofilm structure and subsequent dispersion/reduction in adherent microorganisms, and (iii) damage of extracellular polymeric substance (EPS) components including proteins, extracellular DNA, and polysaccharides (Figure 1B). It has demonstrated three major morphological changes in microbial cells: cell shrinkage, formation of vacuoles, and cell breakage/leakage of intracellular contents. Other structural and functional changes induced by aPDT include membrane permeabilization, leakage of macromolecules such as proteins and nucleic acids, efflux of potassium ions, reduction in membrane potential, and inhibition of respiration (Figure 1C). 

The photoinactivation mechanism of some natural photosensitizers on foodborne bacteria and fungi is roughly the same, but the types of photosensitizers and light sources used may be different. For example, when inactivating bacteria, photosensitizers such as blue light and curcumin are usually used, while when inactivating fungi, it is more likely to combine psoralen and UVA light. Moreover, when inactivating bacteria, photosensitizers need to be absorbed by specific structures of bacteria, while when inactivating fungi, photosensitizers only need to be able to penetrate the cell walls and cell membranes of fungi [6,7]. Although most of the current studies elucidating the mechanism of aPDT are mainly focused on areas such as clinical medicine, theoretical studies targeting foodborne microorganisms are gradually gaining attention; for example, Alessandra Gilda Ritacca et al. [8] considered the relative amounts of different compounds in neutral and ionic forms in water and changes in pH, calculating the absorption properties, spin-orbitals, vertical ionization potentials and vertical electron affinity potentials of each species to investigate the occurrence of Type I and Type II photoreactions, coupling constants (SOC), vertical ionization potentials and vertical electron affinity potentials, and to explore the mechanism of action of monomeric anthraquinones emodin as a photosensitizer against Gram-negative foodborne bacteria (*Acinetobacter baumannii*, *Pseudomonas aeruginosa*, etc.). Micha et al. [9] provided some insights into new mathematical modeling of the mechanics of microbial inactivation during food preservation, including issues such as microbial sterility assessment, mathematical representation of microbial survival as a stochastic process, and the validity of kinetic parameters. aPDT’s novel academic model for food spoilage-causing microorganisms should be based on a model of a completely stochastic Markov chain for further development and testing.

On the one hand, the properties of aPDT, like photosensitizers, affect food preservation outcomes. On the other hand, factors such as food water activity and texture also play significant roles, but we categorize them into different product matrices, which will be discussed in the context of the application of aPDT in different food preservation. Only the influencing factors related to the three elements of aPDT are discussed here.

The choice of light source directly affects the wavelength and dose of light. Considering the economic cost, thermal effect, spectrum width, and other issues, a light-emitting diode (LED) is the optimal choice, as lower cost, less dangerous, and thermally non-destructive; the wavelength range is determined, making it easy to use flexible arrays. Most of the light sources used in aPDT are red, green, and blue [3]. And it has been shown that blue light has the best bacteriostatic effect followed by green light, and red light is the worst [11]. Nguyen Hien Minh et al. [12] compared the effects of blue and red LEDs on the inactivation of *Staphylococcus aureus* and found that the inactivation of *S. aureus* was about 53% when exposed to blue LEDs at a dose of 138.09 J/cm^2^ for 15 min, while on the contrary, the red LEDs led to an increase rather than a decrease in the growth of the bacteria of about 15%; and when the treatment time was longer than 20 min, the red LEDs also led to a significant increase in the air temperature to 36.5 °C, while the blue LEDs kept the temperature below 35 °C. Currently, blue LEDs have been shown to have the ability to inactivate a wide range of bacteria, such as the common foodborne bacterium *S. aureus*. They also demonstrated the dependence of *S. aureus* inactivation on irradiation factors, including time, distance, and energy dosage, and proposed a DNA self-repairing leading to a delayed time in the inactivation process of *S. aureus.* The optimal conditions for complete inactivation of *S. aureus* using blue LEDs were found to be 35 min of irradiation at an energy dose of 322.2 J/cm^2^ at a distance of 5 cm by a one-way experiment. Controlling the wavelength of the light source not only ensures that it is sufficiently penetrating and energetic but also ensures overlap with the absorption wavelength of the photosensitizer for better sterilization. However, it has been proposed that red, blue, or green light alone can maintain the color, texture, and nutrition of freshly cut fruits and vegetables, but with a lower bacteriostatic effect [13]. Therefore, the combined application of multiple light sources and different technologies will be a major trend in the future development of aPDT for food preservation. In addition, controlling the light dose (i.e., light flux, light flux rate, and light irradiation time) on the one hand, it directly affects the effect of aPDT, and on the other hand, it will affect the amount of heat generated, which affects the sterilization effect of aPDT. It has been shown that when the light dose is 5.6 J/cm^2^ (180–1100 nm), the surface temperature of vegetable leaves will increase from 20 °C to 80–100 °C, and the heat effect negatively affects the sterilization effect and food quality. Yuan Yuan et al. [14] investigated the photodynamic inactivation of *S. aureus* by curcumin as a photosensitizer and compared the photodynamic inactivation of *S. aureus* by curcumin with different light doses and found that the best inactivation of *S. aureus* was achieved by 10 μM curcumin at a light dose of 1.296 J/cm^2^, and the modified Gompertz model showed a good fit with their inactivation data for *S. aureus*. The distance of the light source from the food should not be neglected, to ensure the effectiveness of aPDT while avoiding the close proximity that leads to skewed luminous flux distribution and uneven light radiation. Skewing may be avoided by using light arrays (e.g., LED strips) spanning the entire length of the surface, but sometimes this approach is not practical and has to be switched to a single high-intensity light source to enhance the uniformity of the distribution [15]. 

**Figure 2:**
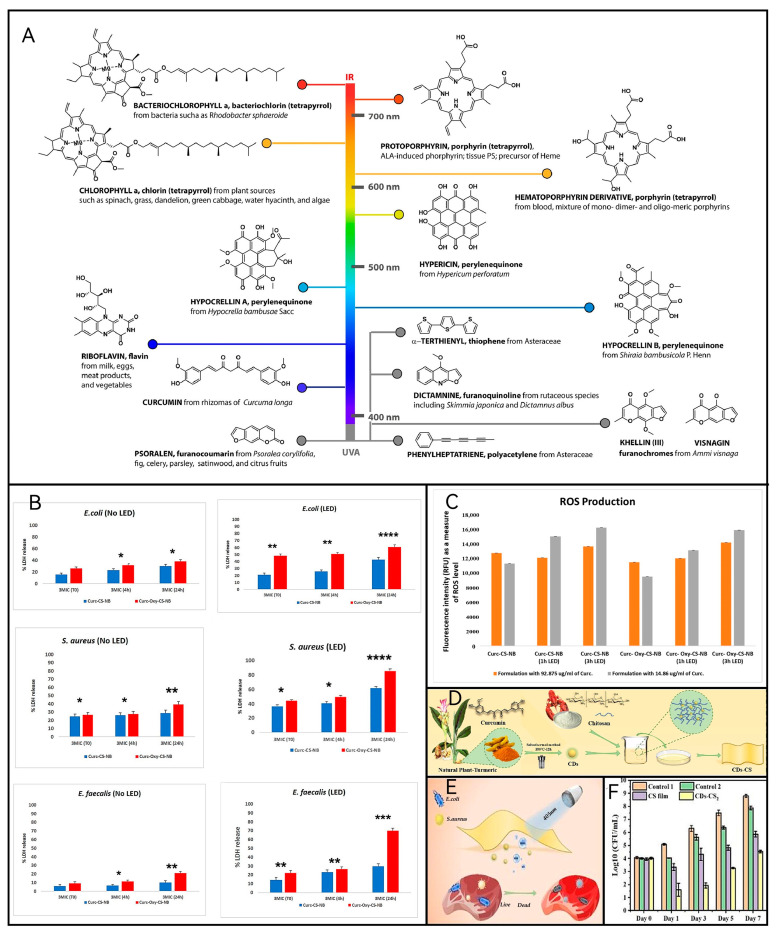
(**A**) Examples of natural PS with therapeutic application [19]. (**B**) Curc-CS-NBs and Curc-oxy-CS-NBs under LED and dark conditions against the tested bacterial strains [20]. (Vs controls: * *p* = 0.0332; ** *p* = 0.0021; *** *p* = 0.0002; **** *p* < 0.0001) (**C**) Reactive oxygen species generation with LED irradiation (0–3 h) in response to high and low concentrations of curcumin in nanoformulations (Curc-CS-NB and Curc-CS-Oxy-NB) [20]. (**D**) Preparation method of CDs-CS [19]. (**E**) Antibacterial strategy [19]. (**F**) The total bacteria count [21].

Oxygen also affects the effect of aPDT, but since oxygen promotes food oxidation but is not conducive to food preservation, almost no research has been made in food preservation to affect the effect of aPDT by modulating oxygen. Only Zunaira Munir et al. discussed the results of the time-kill investigations (Figure 2B) and the situation of ROS production (Figure 2C) by using innovative chitosan-shelled carriers, i.e., curcumin-containing nanobubbles (Curc-CS-NBs) and oxygen-loaded curcumin-containing nanobubbles (Curc-Oxy-CS-NBs). Clinical studies have shown that the inhibitory effect of aPDT on cells under a hypoxic environment is obviously inferior to that when oxygen is sufficient [16]. In the field of food preservation, on the one hand, there are a lot of antioxidants in the food itself, for example, vitamins and β-carotene widely found in fruits and vegetables can protect cell membranes, and vitamin C can eliminate free radicals in the cells, which is undoubtedly a big problem for the application of aPDT in food preservation [17]. On the other hand, some methods of food packaging and storage, such as vacuum packaging and gas-conditioned storage, which are now applied on a large scale commercially, require a low-oxygen or anaerobic environment, which undoubtedly poses a challenge to the application of aPDT in food preservation. Although the research on oxygen is still limited, it is foreseeable that this will be a popular research direction for aPDT for food preservation in the future. 

### 2.2. Photosensitizer

Photosensitizers (PS) play a central role in aPDT, and research into food preservation using various photosensitizers is well established. This includes both endogenous PS found in microorganisms and the addition of exogenous PS. Figure 2A provides examples of natural PSs with therapeutic applications. Of particular note is riboflavin, also known as vitamin B2, which stands out for its potential in food preservation due to its metabolizable nature in the human body. Guangdong Sun et al. [18] enhanced the photoinitiation efficiency of flavin mononucleotide by introducing persulfate. They demonstrated that the combination of flavin mononucleotide (FMN-), a water-soluble derivative of riboflavin, with potassium persulfate exhibited superior photoinitiation efficiencies across various biomaterials. This finding underscores the potential of riboflavin in aPDT applications. Additionally, it is noted that the precursor substance for photosensitizers, 5-amino ketoglutaric acid, is gaining attention, although current research has not extended into the realm of food preservation.

Endogenous PS undergoes a series of biochemical reactions within the organism upon irradiation with specific wavelengths of light. However, optimization and improvement are typically necessary to enhance the efficiency of aPDT for improved food preservation. For example, Cláudia P. S. Ribeiro et al. [22] prepared reversed-phase cationic porphyrin–cyclodextrin couplings and applied these water-soluble porphyrins as PS for the inhibition of *Escherichia coli* activity, finding that the optimal aPDT efficiency was achieved at 5a for each series of methoxypyridinium dyes versus thiopyridinium dyes, with a reduction in bacterial viability of 3.5 log10 (50 mW/cm^2^, 60 min of light exposure), an overall decrease in bacterial viability at 8a (>8 log10, 25 mW/cm^2^, 30 min of light exposure), and a slightly poorer inhibition of *E. coli* in the presence of methoxypyridinium units.

The most widely used exogenous natural photosensitizer is curcumin, which is currently effective in inactivating common foodborne microorganisms such as *Listeria monocytogenes*, *Vibrio parahaemolyticus*, and *Staphylococcus aureus*. As for the synthetic photosensitizers, most of them are organic dyes such as methylene blue and fluorescent yellow, they are usually used in combination with other natural photosensitizers. Dias Lucas D et al. [23] explored the combination of methylene blue and curcumin, finding that simultaneous photoactivation of the two (at 450 nm and 660 nm) induces both synergistic and competing effects instead of an additive process. In addition to competitive rivalry between them (causing photobleaching) and the target bacteria through the formation of ROS and ^1^O_2_, the effects that may result from the simultaneous use of the two photosensitizers on bacteria depend on the attenuation of light by the photosensitizer molecules, the shielding effect and spatial site resistance of the photosensitizers, and inter- and intra-photo-bleaching.

The utilization of exogenous photosensitizers requires further supplementation. Research on developing novel packaging materials with antimicrobial activity has emerged as a significant focus of antimicrobial photodynamic therapy (aPDT) for food preservation, encompassing efforts to identify efficient loading carriers and construct membrane systems. For example, Fangzhou Wen et al. [21] incorporated fluorescent carbon quantum dots (CDs) prepared from the natural plant turmeric into a chitosan matrix to prepare a composite food packaging film (CDs-CS) with good mechanical (Figure 2D), UV protection, and hydrophobicity properties, and the composite film was capable of generating abundant reactive oxygen species when irradiated with a 405 nm light source (Figure 2E), which for 40 min resulted in the reduction of *Staphylococcus aureus* and *E. coli* by about 3.19 and 2.05 log10 CFU/mL, respectively. In pork cold storage application, the total number of bacteria (104.5 CFU/mL) in the CDs-CS2 group on the 7th day of storage was still lower than the maximum acceptable limit (107), and the CDs-CS2 film successfully prolonged the shelf life of pork from 3 days to about 10 days (Figure 2F). Li Nan et al. [24] incorporated curcumin into bacterial cellulose nanofibers/konjac glucan (KGM)-based smart films. The KGM-based smart films containing 3% curcumin exhibited superior freshness preservation performance. These films gradually changed color to redder hues as the packaged beef deteriorated, also functioning effectively as pH colorimetric indicators for meats. Additionally, Zunaira Munir et al. [20] demonstrated that encapsulating curcumin in nanobubbles (NBs) markedly enhanced its solubility and stability. Using curcumin-loaded nanobubbles as innovative carriers resulted in minimal inhibitory concentrations (MICs) against *S. aureus* as low as 12 µg/mL (with oxygen loading) and 46 µg/mL (without additional oxygen loading) following aPDT.

### 2.3. Application of aPDT in Different Food Matrices

Naturally occurring photosensitizers are currently utilized in the food preservation field, including chitosan, chlorophyll, curcumin, and hypericin, with applications predominantly focused on various food matrices such as fruits, vegetables, aquatic products, dairy products, and meat. Table 1 summarizes studies conducted between 1 January 2022 and 30 April 2024, concerning the use of natural photosensitizers for food preservation [25,26,27,28,29,30,31,32,33,34,35,36,37]. In addition, Aliaksandr V. Mikulich et al. [38] conducted a comprehensive investigation into medicinal plant extracts (Calendulae officinalis floridis extract, Chamomillae recutitae floridis extract, Achillea millefolii herbae extract, Hypericum perforatum extract, and Eucalyptus viminalis folia extract) as photosensitizers for aPDT. Their study revealed oxygen singlet yields ranging from 0.40 to 0.64 at different wavelengths under an equivalent energy dose of 30 J/cm^2^ following photodynamic sterilization treatment (irradiation at 100 mW/cm^2^ for 5 min). This treatment significantly reduced both *Pseudomonas aeruginosa* and *S. aureus* counts by >3 log10 and 4 log10, respectively.

#### 2.3.1. Fruit and Vegetables

Fruit and vegetable products are popular because they are rich in vitamins and organic acids. However, fresh fruits and vegetables are still undergoing respiration-based metabolic activities after picking, and by continuously consuming carbohydrates, vitamins, organic acids, and other substances within the fruit to carry out metabolic activities, resulting in a decline in the quality of fruits and vegetables. In addition, the high moisture and sugar content of fruits and vegetables are also highly susceptible to the growth of corrosive microorganisms, which shorten the shelf life of fruit and vegetable products. At present, aPDT in the preservation of fruits and vegetables has been a lot of research, for example, Yuchen Zhang et al. [34] using chlorophyll (1 × 10^−5^ mol/L) and 405 nm light (22.27 J/cm^2^/day) on fresh-cut pakchoi aPDT, observation of fresh-cut pakchoi storage during the quality of the changes, the results show that aPDT-treated pakchoi had much higher visual quality than other pakchoi, and the contents of chlorophyll, VC, total soluble solids, and SOD activity were significantly increased, and the occurrence of leaf yellowing and POD activity was reduced. Ilaria Stura et al. [39] sprayed berries such as strawberries and blueberries with two different concentrations of curcumin (0.5 mg/mL and 1 mg/mL), and both were subjected to 3 h of blue LED irradiation for oxidative and quantitative assays and found that the treatment of light-activated curcumin solution reduced the number of bacteria (from 3.1 to 2.5 UFC/mL) and did not alter the fruit’s organoleptic and antioxidant properties. Ting Du et al. [40] used edible coatings (pectin/quercetin) derived from the FDA for up to 10 days while preserving as much as possible the color of the apples, maintaining 100% firmness, 80% of the sugar content, and 17.3% weight loss, achieving shelf life and protecting the commercial quality of fresh-cut apples. Yingbin Lv et al. [41] constructed gelatin–sodium carboxymethylcellulose composite films loaded with curcumin to preserve fruits and found that films containing 0.5% Cur had the highest antibacterial rate against *Staphylococcus aureus* and also achieved 95% antibacterial rate against *Escherichia coli*. Grapes had a shelf life of up to 9 days after light treatment with the film, and the microbial content of the skin was much lower than that of the control group; bananas exposed to the film-forming solution for a short period of time stayed fresh for 6 days, and the weight loss was significantly slower than that of the control group. In addition to freshly cut fruits and vegetables such as chard and apples, a considerable number of studies have used fruit and vegetable juices as substrates, such as some of the studies in Table 1, which used carrot juice [29], white grape juice [32], and apple juice [33,37] as substrates.

#### 2.3.2. Seafood

Aquatic products are delicious, high nutritional value; with the improvement of the economic level and the development of preservation and transportation technology, the consumption of aquatic products, especially raw food aquatic products, has increased greatly. However, since aquatic products are rich in protein and water, they are susceptible to bacterial growth, spoilage, and deterioration during transportation or after inactivation after being removed from their original living environment and are not easy to store. In addition to the studies on raw fish slices summarized in Table 1, the related preservation studies on salmon are quite sufficient. Qiao-Hui Zeng et al. [42] treated salmon stored at 4 °C with curcumin under irradiation of 18.72 J/cm^2^ and effectively inactivated the bacteria by 3.0 log10 CFU/g (99.9%). Additionally, they found that, compared with the control group, aPDT inhibited protein degradation and reduced protein degradation by 7.7% and also reduced the oxidation of unsaturated fatty acids by 21%, maintaining the color and pH of salmon, preserving the integrity of muscle fibers, and reducing water loss during low-temperature storage. Jiaming Huang et al. [43] treated *Listeria monocytogenes*-contaminated salmon with curcumin and a blue light-emitting diode (LED), and compared to the negative control, the aPDT effectively slowed down the proliferation of *Listeria monocytogenes* in salmon during storage at 25 °C, with a maximum inhibition of 4.0 log10 CFU/g (99.99%), delayed the increase in pH and the production of TVB-N, the accumulation of free fatty acids, slowed down the degradation of proteins, and ultimately retained the high nutritional value of the salmon. It also effectively prevented salmon color change and water loss and maintained its texture and sensory properties. Lu Chen et al. [44] investigated the aPDT-mediated preservation effects of poly(lactic acid)/5-aminolevulinic acid (PLA/ALA) films on salmon fillets’ storage quality under the irradiation of blue LEDs (20 W, 455–460 nm) to inhibit microbial activity-induced food spoilage and protein degradation, while also preserving salmon protein concentration, spatial structure, and salmon muscle integrity. More importantly, the PLA/ALA film consumed oxygen to produce ^1^O_2_, which directly mitigated the lipid oxidation process. This method provides an excellent idea for preserving aquatic products.

#### 2.3.3. Dairy Products

Dairy products are usually pasteurized (low-temperature sterilization at around 72–85 °C ) and are therefore highly susceptible to the growth of spoilage bacteria, such as *Escherichia coli* O157:H7, *Staphylococcus aureus*, and *Listeria monocytogenes*. The inactivation of these foodborne microorganisms by aPDT has also been investigated, in particular, the photodynamic inactivation study of *Pseudomonas fluorescens*, *Listeria monocytogenes*, and *Escherichia coli* in milk. aPDT is also a powerful and efficient method for the inactivation of foodborne microorganisms in milk. Juliana Beatriz Miazaki et al. [45] applied a combination of coating and aPDT in the preservation of whey cheese. They divided the samples into three groups (cheese samples coated with alginate solution, alginate solution, and 10 μmol/L erythrosine) and illuminated the three arrays of samples with a green LED lamp for 15 min (510 nm; 10 mW/cm^2^); Salmonella and *Staphylococcus aureus* were not detected in any of the three groups during the 21-day storage time, but a 1 log cycle reduction in coliform counts was observed only at 100 μmol/L erythrosine coating, and none of the three groups of samples were induced to oxidize by aPDT. Bruna Barnei Saraiva et al. 2021 [46] used dimethyl sulfoxide or ethanol as solubilizers, exposed *Pseudomonas fluorescens* in Minas Frescal cheese in vitro for 5 min and in situ for 30 min under blue light emitting diode (450 nm, 2.7 mW/cm^2^) irradiation, and found that 62.50 μg/mL of curcumin inactivated 7 log10 CFU/mL of *Pseudomonas fluorescens* and maximized cheese hardness and chewability and reduced protein hydrolysis. They also inactivated *Pseudomonas fluorescens* in milk using a blue light-emitting diode (2.7 mW/cm^2^, with a total energy dose of 0.81 J/cm^2^ applied during a 5 min exposure) in 2023 [46] and found that although the milky white matrix impeded the absorption of light by the photosensitive molecules (PS), there was still significant inhibition of *Pseudomonas fluorescens* (7 log10 CFU/mL) and that macronutrient composition and protein oxidation levels were not altered. However, it is clear that the problems of light-induced lipid peroxidation and PS changing the color of milk limit the application of aPDT.

#### 2.3.4. Meat Products

Meat products are prosperous in range and are a good source of protein for the human body and are rich in iron, zinc, and different critical hint elements. However, if they are not correctly preserved, they can be easily contaminated by microorganisms such as Staphylococcus aureus, Salmonella, and E. coli, and traditional frozen storage can produce ice crystals, which can affect the quality of meat products. Therefore, photodynamic science has long been utilized in the preservation of meat products. Shengyu Zhu et al. [34] studied the renovation of pork in vitro and underneath D65 LED (400–800 nm) irradiation by means of applying aPDT to erythrosin B (EB) loaded in corn starch (CS) films on foodborne microorganisms such as *S. aureus*, *E. coli*, and *Salmonella*, evaluation revealed that CS-EB films produced 26.36 μg/mL of hydrogen peroxide and 74.5 μg/g of hydroxyl radicals underneath irradiation, and the inhibition of bacterial boom was once 4.7 log10 CFU/mL in the 5% EB-CS group after 30 min of in vitro mild exposure and 2.4 log10 CFU/mL on pork samples. Jingru Wu et al. [30] used κ-carrageenan as a film-forming substrate loaded with curcumin-β-cyclodextrin (Cur-β-CD) complicated to put together a biodegradable photodynamic antimicrobial film by using the casting method, which was used for chilled pork (refrigerated at 4 °C) and subjected to aPDT (425 nm, 45 min), and it was found that the shelf life was extended from 4–5 days to 10 days. After 10 days of storage, the bacterial count on pork has only reached 5.78 ± 0.17 log10 CFU/g, the complete unstable alkaline nitrogen content has only reached 12.35 ± 0.57 mg/100 g, and the average sensory evaluations including color, odor, and water-holding ability were better than those of the control group.

## 3. Antibacterial and Food Preservation Based on Ionizing Radiation

Since aPDT is influenced by the absorption wavelength of the photosensitizer, its primary light source is visible light. However, shortening the wavelength enhances its sterilization efficacy, potentially enabling sterilization directly through ionizing radiation without relying on photosensitizers.

Ionizing radiation sterilization, commonly referred to as food irradiation, is a non-thermal technique for preserving food. It involves applying specific doses of ionizing radiation, which includes electromagnetic gamma (γ) rays emitted by radioactive cobalt (60 Co) or cesium (137 Cs), as well as β-rays generated from electron emissions at the cathode. This method is utilized to inhibit certain physiological processes like germination and ripening in fresh food, thereby sterilizing it to extend shelf life and enhance food quality [47]. Since 1983, the Codex Alimentarius Commission of the United Nations has acknowledged that foods irradiated below a specific threshold (10 kGy) remain safe and nutritious. Consequently, food irradiation is no longer categorized as a food additive but is recognized as a legitimate food processing technique.

The use of ionizing radiation for sterilizing and preserving processed foods is now well-established and strictly regulated. Gamma radiation has garnered extensive experimental validation, whereas electron beams and X-rays have seen comparatively less application. Numerous studies demonstrate gamma radiation as an effective method for preserving foods by deactivating microorganisms and prolonging shelf life. However, practical challenges in utilizing radioisotopes have hindered the commercialization of gamma-ray irradiation, whereas electron beam treatment shows greater promise in achieving widespread adoption for food safety [48]. Sterilization via ionizing radiation offers significant energy savings since it does not necessitate heating the food product. This method can be applied to frozen or chilled products without causing substantial thawing due to minimal temperature elevation. Furthermore, irradiation enhances the hygiene and safety of processed foods, facilitating their storage and distribution [49]. Consequently, this part will focus on exploring the factors and specific studies examining the impact of ionizing radiation on preserving various types of food products.

### 3.1. Mechanism of Ionizing Radiation

**Figure 3:**
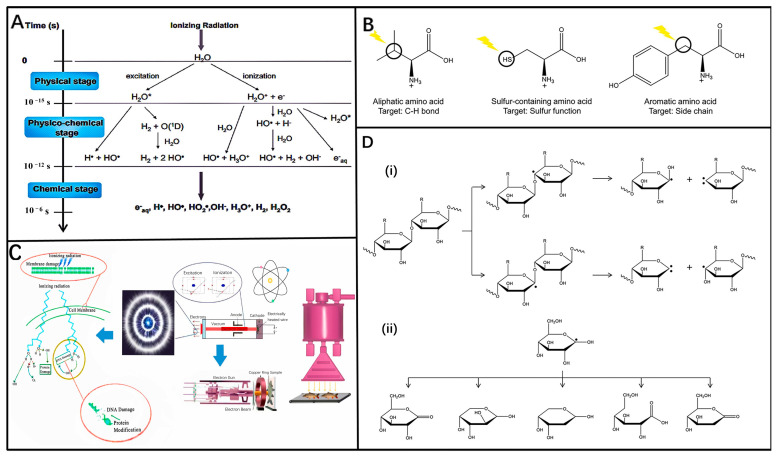
(**A**) The three phases of water radiolysis’ primary reactions [55]. (**B)** Effect of ionizing radiation on amino acids [56]. (**C**) The mechanism of electron beam irradiation generation, and the inactivation mechanism of ion-pair to cell’s DNA [57]. (**D**) Representative examples demonstrating the effect of ionizing radiation on polysaccharides: breakage of glycosidic bonds and free radical reactions of D-glucose (**i**) Breakage of glycosidic bond by ionizing radiation. (**ii**) Examples of free radical reactions of D-glucose [56].

**Figure 4:**
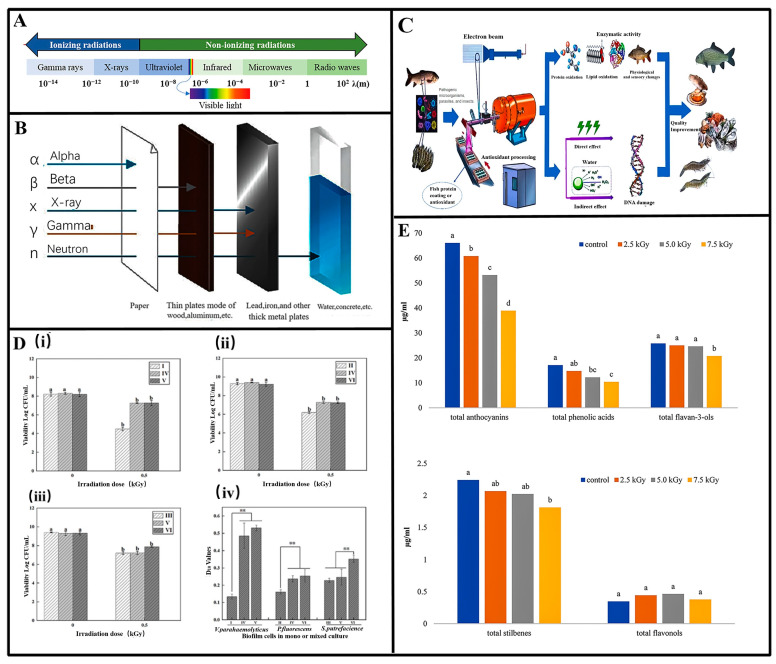
(**A**) Electromagnetic spectrum [62]. (**B**) Type of ionizing radiations and penetration depth [63]. (**C**) The process of seafood sterilization through electron beam irradiation [57]. (**D**) Count and D10 values of biofilm cells treated by electron beam irradiation. (**i**–**iii**) The bacterial counts of *V. parahaemolyticus*, *P. fluorescens*, and *S. putrefaciens* after irradiation (0.5 kGy) under mono-culture and co-culture, respectively, and (**iv**) the same under mono-culture and mixed-culture treated by electron beam irradiation, respectively (Lowercase letters indicate a significant difference, ** *p* < 0.01) [64]. (**E**) Content of polyphenolic compounds in relation to the ionizing radiation dose (Lowercase letters indicate homogeneous groups within a group of polyphenolic compounds) [65].

Ionizing radiation, characterized by its short wavelength and high intensity, can deeply penetrate the food matrix and induce ion formation within atoms. Its impact on food can be categorized into primary and secondary effects. The primary effect involves direct irradiation by high-energy electron rays of microbial cells, lipids, carbohydrates, and amino acids, leading to ionization and chemical alterations (Figure 3B–D). The secondary effect occurs in the presence of water (Figure 3A), where radiation and ionization generate various reactive substances like free radicals (e.g., electrons, hydrogen atoms, hydroxyl radicals), which interact with cellular components. These actions collectively disrupt microbial cellular activities, ultimately causing cell death [50]. The physicochemical effects of food irradiation closely align with the mechanisms of high-energy rays. When these rays encounter atoms in irradiated materials, they induce processes such as ionization, Compton scattering, annihilation radiation (electron pair effect), and sensitized radiation, influencing the behavior of foodborne microorganisms and aiding food preservation [47] (Figure 4E). However, ionizing radiation also unavoidably alters food components such as moisture, proteins, enzymes, sugars, lipids, and vitamins, thereby impacting the sensory attributes like flavor and texture of the food.

Gamma rays and X-rays possess sufficient energy to cause ionization and share similar penetrating capabilities. However, X-rays originate from electrons located outside the nucleus, whereas gamma rays originate from within the nucleus. Several studies explore both the similarities and distinctions among these forms of ionizing radiation. For instance, Kroc Thomas K. conducted computer simulations to examine their relationships and utilized Monte Carlo simulations to illustrate that below 500 keV, there are minimal differences in the energy spectra of gamma rays from cobalt-60, MeV X-rays, and MeV electrons. This indicates that the doses delivered by these three types of ionizing radiation are essentially equivalent [51]. Payman Rafiepour et al. [52] conducted multiscale Monte Carlo simulations to compare the dose distributions of low-energy X-rays and electrons in a typical-sized apple, while also evaluating secondary electron-induced DNA damage in a bacterial cell model. The study revealed that at the microscale, the induction of single- and double-stranded DNA breaks by X-rays and electrons of varying energies was nearly identical. At the macroscale, a double-sided electron beam configuration, combined with a 90-degree rotation of the apple relative to the beam, achieved uniform dose distribution to a depth of up to 40 μm on the apple’s surface. In contrast, low-energy X-rays provided uniform dose distribution on the apple’s surface without requiring rotation, highlighting their superiority over electrons for apple surface treatment (Figure 4A). Among the three types of radiation discussed, gamma rays stand out as the most extensively studied and commonly employed. Characterized by a short wavelength (0.001–0.0001 nm), strong penetration capability, and extensive range, gamma rays are highly effective in eradicating microorganisms. For instance, when applied to various vegetables contaminated with *E. coli*, simultaneous exposure to gamma irradiation (1.5 kGy) and E. coli phage treatment resulted in a remarkable reduction in bacterial counts exceeding 99.99% within 24 h. This dual approach also proved effective in reducing polysaccharide concentration and protein within the biofilm matrix. Experimental findings underscored that the combined use of gamma irradiation and E. coli phage was more potent in disrupting the biofilm matrix compared to either treatment alone [53]. And gamma radiation can irradiate a lot of material at a time with a relatively uniform dose. However, it is dangerous and must be shielded (a few cm of lead plates or a concrete wall several meters thick) (Figure 4B). In contrast, X-rays exhibit relatively limited penetration capabilities, making them suitable for surface sterilization. They are effective in inhibiting the germination of garlic and potatoes and can also be utilized for sterilizing spices. Son Nguyen An et al. [54] examined the impact of low-energy (160 keV) X-rays on microbial inactivation, germination inhibition, and genetic variation in potatoes. Their findings indicated that X-ray exposure reduced the microbial population in potatoes to 0.59% at a dose of 1000 Gy and a dose rate of 13.87 Gy/min. Unirradiated potatoes exhibited spontaneous sprouting within 2 months, whereas those irradiated at 1000 Gy maintained their quality for up to 6 months. Furthermore, the degree of DNA structural variability in potatoes was minimized at an irradiation dose rate of 13.87 Gy/min. By these three aspects, X-ray treatment can effectively extend the shelf life of potatoes.

The purpose of food radiation sterilization is different, and the radiation dose used is also different: The irradiation dose of complete sterilization is 25–50 kGy, and its purpose is to kill all microorganisms except bacillus; the radiation dose of disinfection and sterilization is 1–10 kGy, and it can kill pathogens that do not produce bacillus and reduce microbial contamination of the food in order to prolong the shelf life [58]. Furthermore, Figure 4D shows the count and D10 values of different biofilm cells treated by electron beam irradiation. It is also necessary to determine the type of radiation to be used according to the actual food situation and safety measures.

In the ambient temperature range, temperature has little effect on the sterilizing effect of irradiation. However, irradiation at low temperatures reduces the activity of free radicals generated during radiation and reduces the fracture and decomposition of food components in order to prevent oxidation of food components and thus maintain the quality of the food [51]. The results of the study showed that gamma-ray irradiation treatment of Clostridium botulinum’s spores had no effect of temperature on the bactericidal effect in the range of 0–65 °C; in the range of 0–196 °C, the lower the temperature, the greater its resistance to radiation. However, from the view of the effect of killing microorganisms alone, the temperature of irradiation has little effect. The influence of temperature during irradiation is considered because temperature can affect the taste and quality of food. For meat, poultry, and other protein-rich animal foods, low-temperature ionizing radiation treatment can reduce the denaturation of proteins. A large number of studies have shown that quick-frozen treatment of animal foods in the range of −40 to −8 °C radiation is the best, which can ensure the maximum possible food quality.For fruits and vegetables, the irradiation at −75 °C minimizes vitamin loss. For example, vitamin C in frozen orange juice was preserved even at a 9 kGy dose of irradiation at −75 °C [57]. Therefore, ionizing radiation at low temperatures can ensure that the quality and nutrient content of foodstuffs do not change as much as possible under the condition of ensuring the preservation effect.

The oxygen level in food packaging significantly impacts the efficacy of ionizing radiation for sterilization, typically more effective in aerobic environments. Bacterial spores, when exposed to gamma rays or electron beam irradiation, show greater susceptibility in air compared to vacuum or nitrogen environments. However, radiation can ionize oxygen in the air, producing ozone that oxidizes proteins and fats in food. High-protein and high-fat foods benefit from vacuum sealing or nitrogen flushing to mitigate this effect. Alternatively, irradiation in a nitrogen-filled environment can prevent vitamin loss. Combining ionizing radiation with free radical scavengers, sensitizers, or other preservation methods synergistically enhances food preservation and compensates for reduced effectiveness under low oxygen or vacuum conditions.

### 3.2. Applications of Ionizing Radiation in Different Food

The impact of ionizing radiation on different types of food varies due to factors such as the food’s chemical composition, structure, growth stage, maturity, cellular respiration rate, metabolism, and the presence and extent of microbial contamination.

Foods with higher moisture content tend to absorb ionizing radiation more readily, potentially achieving better sterilization at the same dose. However, excessive moisture can also lead to softening or liquefaction of the food post-irradiation, altering its texture and taste [51]. Fiber content, particle size, and shape influence structural densification, with a tight structure potentially impeding radiation distribution, while a loose structure aids deeper penetration and enhances treatment efficacy. The acidic environment also impacts sterilization: A lower pH weakens microorganism cell walls and membranes, facilitating internal destruction by ionizing radiation for improved sterilization. Additionally, acidity can alter microbial metabolism, lowering resistance to ionizing radiation. Given the unique challenges of marine environments, seafood is particularly vulnerable to microbial contamination, prompting extensive research into ionizing radiation for sterilization purposes (Figure 4C)**.** Muhammad Majid Noor et al. [59] studied the preservation of shrimp cakes using gamma rays (3 kGy and 5 kGy) and Total Asparagus Bread Powder (TAB) ARP (3%). Samples treated with 5 kGy + 3% ARP showed complete bacteria inactivation throughout storage days (0, 7, 14, 21) and eliminated coliform bacteria. TAB resulted in full bacterial inactivation in shrimp cakes treated with 5 kGy + 3% ARP. Moreover, MMb levels decreased in shrimp cakes treated with radiation and 3% ARP but increased in those treated with radiation alone, suggesting ARP effectively inhibited radiation-induced hemolysis. The antioxidant value (DPPH) of shrimp cakes decreased with increased γ irradiation and over time, indicating low-dose ionizing radiation can extend shrimp cake shelf life. Hai-lan Li et al. [60] used cobalt source γ-ray irradiation to assess the preservation of crayfish. Results showed that as the irradiation dose increased, total colony counts in crayfish meat significantly decreased, while the total volatile base nitrogen (TVB-N) value increased significantly (*p* < 0.05). At an ionizing radiation dose of 3.32 kGy, both sterilization and optimization of crayfish meat quality were achieved. Combining gamma irradiation with low-temperature storage at 0 °C extended the shelf life of crayfish to 9 days. In addition, Fengqi Wang et al. [61] explored how non-thermal processing techniques, such as ionizing radiation, impact the structure and allergenic properties of seafood allergens. These methods disrupt the structural integrity of seafood allergens by breaking their covalent and non-covalent bonds. This process leads to degradation, aggregation, unfolding, and exposure behaviors in allergenic proteins, resulting in significant conformational changes that either cover or expose original epitopes. As a result, the sensitizing potency of these allergens is affected. Importantly, the structural alterations induced by non-thermal treatments also affect the allergens’ susceptibility to digestive enzymes, particularly pepsin, thereby influencing food digestion and absorption.

Ionizing radiation is also of wide interest in the preservation of fruits and vegetables. This is because, in addition to eliminating spoilage-causing microorganisms, exposure of fruit and vegetable foodstuffs to controlled doses of ionizing radiation can also prolong shelf life by achieving delayed ripening and senescence of fruits and inhibiting the germination of tubers, bulbs, and other rhizomes [66]. Armarynette Berrios-Rodriguez et al. [67] irradiated carrots and tomatoes with gamma rays, and Salmonella populations in carrots were reduced by 4.3 log10 (5 °C ) and 3.7 log10 (20 °C ) at 1 kGy treatment; in tomatoes for 5.6 log10 and 5.8 log10, respectively, at 5 °C and 20 °C. Salmonella populations decreased linearly with increasing radiation dose. At the same time, they considered the damage and repair of ionizing radiation on carrots and tomatoes: The level of damaged cells was 4.4% and 4.0% for carrots and tomatoes, respectively, at day 7 (5 °C ) of storage, which was lower than that of the samples treated on days 0 and 3, suggesting that the damaged cells of fruits and vegetables are gradually repairing themselves after being exposed to ionizing radiation. A year ago, they [68] investigated the effect of gamma radiation on the inactivation of *Listeria monocytogenes* in carrots and tomatoes and found similar results and evaluated the effects of gamma radiation (0.5 kGy), lactobacillus peptide-based disinfectant (100%), and competing biocontrol microorganisms (nonpathogenic *Pseudomonas fluorescens*) on the survival of *Listeria monocytogenes* in tomatoes and carrots after harvest at 5 °C and 20 °C and reduced Listeria survival by 2.5–5.0 and 2.4–5.0 log10 CFU/g after day 7 under conditions where all three treatments were implemented simultaneously, respectively. The preservation of wine was studied by Magdalena Błaszak et al. [65]. They found that ionizing radiation eliminated yeast from wine, and that a dose of 2.5 kGy reduced the amount of yeast by more than 90% without degrading the quality of the wine. It was found that, of the yeast species studied, only the *Saccharomyces cerevisiae strain ES181* showed relatively higher resistance to radiation doses of 1 and 2.5 kGy, with all other yeast species beginning to decrease at 1 kGy, respectively.

Ionizing radiation also demonstrates enhanced antimicrobial and antioxidant effects in certain plant-derived foods like propolis and grains. Propolis, composed of resinous substances gathered by bees from diverse plant materials such as tree buds, leaves, bark, and sap, was studied for its response to gamma irradiation. Ralitsa B. Mladenova et al. [69] experimentally confirmed the impact of gamma irradiation on propolis, specifically its polyphenol content and antioxidant properties. Under gamma irradiation, various polyphenols in propolis undergo changes in their scavenging activities. Silymarinic acid levels decrease, and catechins are absent in irradiated propolis. The impact varies with dose: Propolis treated with 2 kGy and 10 kGy shows reduced total phenolic and flavonoid contents, while no changes are observed at 5 kGy. Interestingly, Mladenova et al. found increased phenolic content and antioxidant activity in irradiated honey. Additionally, the use of ionizing radiation to deactivate Enterobacteriaceae in stored wheat was pioneered by Marija Boshevska et al. [70], highlighting its significant implications for the food industry. After irradiation, there was an immediate 1.6 log10 CFU/g (35.6%) reduction in the total microbial population, with significant microbial suppression observed at 5 kGy. *Bacillus cereus* counts were notably reduced to 3.17 log10 CFU/g and 2.3 log10 CFU/g at lower doses of 0.2 kGy and 0.5 kGy, respectively, and completely inhibited at 1 kGy (approximately 3 log10 CFU/g reduction). Additionally, *Salmonella* spp., *coagulase-positive staphylococci*, *Pseudomonas aeruginosa*, *Escherichia coli*, and *Clostridium perfringens* were undetectable post-irradiation, while other microorganisms showed significant reduction after irradiation and nine months of storage. A 1 log10 CFU/g (22.37%) reduction was achieved at 1 kGy, and no live microbial growth was observed in samples irradiated at 5 kGy.

In the case of meat products, ionizing radiation can also have a good preservation effect. For example, the treatment of chicken samples with low-energy electron beams (LEEB) can prolong the shelf life of chicken products. R.A. Vazirov et al. [71] utilized a URT-1 gas pedal to produce nanosecond electron beams (NEBs) with energies below 1 MeV for irradiating chicken samples. The findings indicated that these low-energy electrons effectively treated shallow surface layers, penetrating only up to 1–2 mm in depth. Significant reduction in chicken meat contamination, by 1–1.5 logarithmic units, and potential elimination of pathogenic microorganisms from the surface were achieved with doses up to 10 kGy. Despite the high dose (10 kGy) and structural changes in the surface layer, the nutritional value of the product remained largely unaffected. Asrar Nabil Damdam et al. [72] studied the effectiveness of UV-C irradiation combined with vacuum sealing to prevent microbial spoilage in beef, chicken, and salmon fillets. They sterilized samples using a constant UV-C irradiation dose of 360 J/m^2^ and stored them at a reduced pressure of 40 kPa and found that the use of UV-C irradiation and vacuum sealing increased the meat’s average shelf life by 66%.

## 4. Antibacterial and Food Preservation Based on AMPs

Typical physical techniques such as ionizing radiation penetrate mainly through short waves, while some biomolecules, due to their better fusion effect with microorganisms and food matrices, allow for better penetration effects.

**Figure 5:**
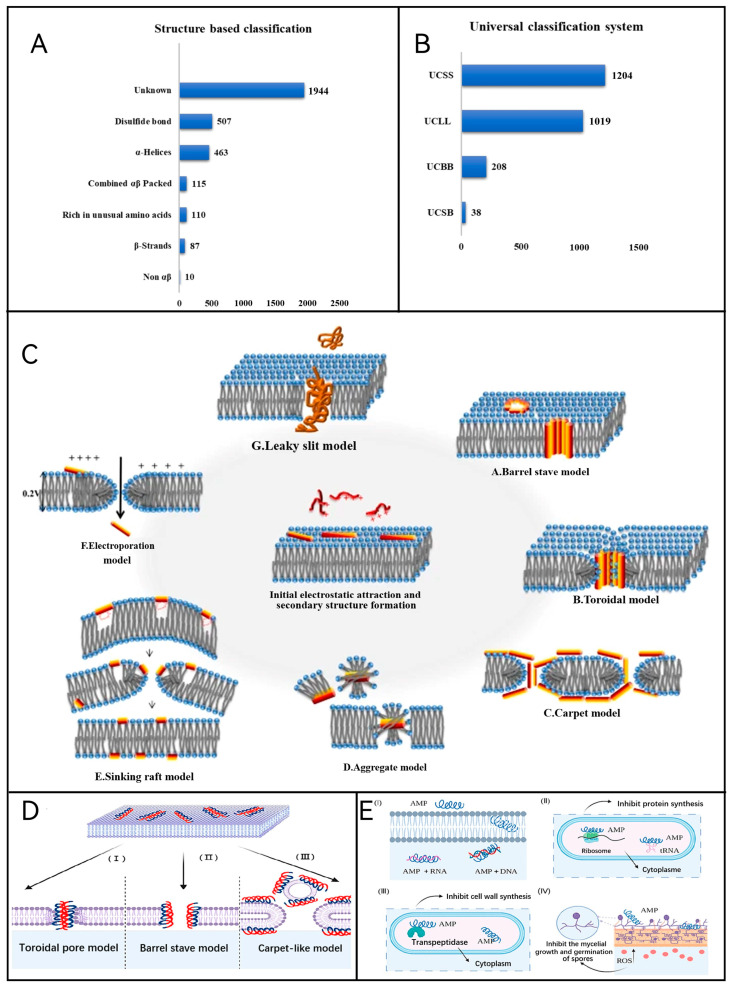
(**A**) Number of AMP 3D structures [74]. (**B**) The AMP universal classification system [74]. (**C**) Mechanism of action of the antimicrobial peptides by membrane lytic mechanism [73]. (**D**) Membrane damage mechanism of AMPs: (**I**) toroidal pore model, (**II**) barrel-stave model, and (**III**) carpet-like model [79]. (**E**) Non-membrane damage mechanism of AMPs: (**I**) Bind RNA and DNA, (**II**) inhibit bacterial protein synthesis, (**III**) inhibit cell wall synthesis, and (**IV**) stimulate ROS overproduction [79].

AMPs are active small molecule peptides that can be produced by all organisms and are characterized by the absence of highly conserved sequences, most of them are very short, amphiphilic, highly cationic, etc. In addition, AMPs have broad-spectrum antibacterial activity against bacteria, especially against certain drug-resistant pathogens. In addition, AMPs have broad-spectrum antimicrobial activity, with a strong killing effect on bacteria, especially their killing effect on some drug-resistant pathogenic bacteria, which has attracted more attention. Figure 5A,B show the number of AMP 3D structures and the AMP universal classification system, respectively. At present, certain antimicrobial peptides are effective in killing some viruses, fungi, protozoa, and cancer cells, which have been widely studied in the field of medicine and gradually emphasized in animal husbandry. In the food field, due to the misuse of pesticides and other foodborne bacteria such as Salmonella, E. aureus, and other foodborne bacteria, there has been a certain degree of resistance developed; hence, some research has begun to draw on the application of antimicrobial peptides in the field of medical science, trying to seek a new method of food preservation based on antimicrobial peptides, mainly focusing on the food additives and preservation film.

Since the discovery of antimicrobial peptides, a great deal of research has been carried out at home and abroad on the mechanism of action of antimicrobial peptides. It is now known that antimicrobial peptides act on bacterial cell membranes, and based on this, a variety of models for the action of antimicrobial peptides with cell membranes have been proposed, such as the carpet model, the polymerization model, and the barrel plate model [73,74] (Figure 5C).

However, strictly speaking, the specific mechanism by which antimicrobial peptides kill bacteria has not been fully clarified yet. It is generally believed that antimicrobial peptides act on the cell membrane (Figure 5D), forming transmembrane ion channels on the membrane, destroying the integrity of the membrane, and making the inner and outer cell barriers disappear, thus killing the cells. However, the specific process of its action, whether there is a specific membrane receptor, there are other factors such as synergistic issues not yet very clear [75]. In addition, antimicrobial peptides can induce intracellular metabolic arrest through targeted inhibition of nucleic acid and protein synthesis, protein folding, inhibition of cell wall synthesis, inhibition of protease activity, and cytokinesis to achieve antibacterial and antimicrobial effects [76] (Figure 5E). Alternatively, they can inhibit protein and nucleic acid synthesis by generating reactive oxygen species and induce apoptosis to exert antimicrobial activity [77]. The redox state of antimicrobial peptides may also drive membrane-independent killing mechanisms, such as the formation of oligomeric structures or ion chelation [78]. The mechanisms of action of other classes of antimicrobial peptides also remain to be further investigated.

### 4.1. Sources of Antimicrobial Peptides

#### 4.1.1. Naturally Occurring

Antimicrobial peptides can be produced in almost all living organisms, from bacteria and fungi to mammals.

Antimicrobial peptides extracted from bacteria, also known as bacteriocins, are a class of peptide molecules or precursor peptide molecules with bactericidal bioactivity generated by bacterial synthesis of proteins, which can be categorized into class I bacteriocins, class II bacteriocins, and class III bacteriocins, among which class II bacteriocins are also known as lactic acid bacteriocins, which are produced by lactic acid bacteria and are currently being paid much attention in the field of food preservation. Insect antimicrobial peptides, of which there are currently more than 200, are a class of peptides produced by hemolymph or related immune sites of the body when insects are stimulated by the external environment. These peptides are classified into five types according to their antimicrobial mechanisms and amino acid sequence compositions: cecropins, lysoalcohol, defensins, glycine-rich polypeptides, and proline-rich polypeptides.

Antimicrobial peptides in marine organisms are the most widely studied. Blind eel intestinal antimicrobial peptide, Hepcidin, and Piscidin found in marine fishes have broad-spectrum inhibitory effects on Staphylococcus yellows, Shigella sonnei, etc.; Shrimpin, which is widely found in various shrimps, selectively targets Gram-positive bacteria and effectively inhibits bacterial activity. Anti-lipopolysaccharide factors (ALFs) are widely distributed in crabs, including PtALF and SsALF, which can selectively inhibit Gram-negative bacteria. Shellfish can also produce a variety of antimicrobial peptides, such as Myticin and mussel defensins (MGD). It has been found that Myticin has significant bacteriolytic activity against Gram-positive bacteria. In addition, there are also a variety of antimicrobial peptides extracted from marine organisms such as jellyfish and sea cucumbers, which have been shown to have broad-spectrum activity against a wide range of microorganisms [80].

In addition, highly effective antimicrobial peptides can be extracted from plants. Currently, different studies have successively identified, characterized, and purified different PAMPs with antimicrobial activity from roots, stems, leaves, flowers, and seeds, such as thionins, defensins, snakins, and lipid-transfer proteins. They have good bactericidal activity against bacteria, fungi, viruses, and parasites. Xudong Gao et al. [81] used Sephadex chromatography, pre-HPLC, and LC-MS/MS techniques to isolate and identify three antimicrobial peptide fractions (F3-3-a, F3-3-b, and F3-3-c) from garlic, and through a series of experiments, it was found that F3-3-c exhibited the strongest antimicrobial effect, with MIC of 100 μM against *Escherichia coli* and *Staphylococcus aureus* bacterial hyphae, with negligible hemolytic activity, and analyzing the structure revealed the presence of α-helix, β-strand, and disordered conformations.

#### 4.1.2. Molecular Modification

Natural antimicrobial peptides are derived from a diverse array of sources and species; however, many of these peptides exhibit toxicity, hemolytic properties, and low activity. Therefore, the modification of antimicrobial peptides is crucial. Currently, the primary methods of modifying antimicrobial peptides involve biochemical and chemical approaches, such as residue substitution, truncation, and the construction of hybrid peptides [82]. For AMPs with known sequences, these modifications typically entail altering the sequence or adjusting the types of amino acids. Such structural changes can significantly enhance their functional properties, including increased antimicrobial activity, strengthened anticancer capabilities, higher expression levels, and reduced hemolytic activity.

Currently, hybrid peptides are one of the most widely used methods in the molecular modification of antimicrobial peptides. Researchers have drawn on protein engineering and alcohol engineering to produce hybrid proteins and hybrid alcohols, and by analyzing the physicochemical properties and activities of different antimicrobial peptides, they recombine selected parts of two different antimicrobial peptides by recombination to form a new hybrid antimicrobial peptide, which can lead to a hybrid peptide with fewer toxicities and higher activity. Huihui Xu et al. [83] designed and constructed a new heterotrimeric peptide, T-catesbeianin-1, by fusing the FyuA-binding domain of pesticin to the N-terminus of catesbeianin-1 through the introduction of a flexible junction (GGGGS)_3_, which was found to be effective against Streptococcus suis, *Bacillus cereus*, *Listeria monocytogenes*, *Escherichia coli*, *Klebsiella pneumoniae*, and *Pseudomonas aeruginosa*; *Klebsiella* and *Pseudomonas aeruginosa* with MICs of 8.83, 4.42, 1.10, 0.55, 1.10, and 1.10 μM, respectively; the lowest inhibitory concentration against most *E. coli* isolates was lower than 1.10 μM. In addition, T-catesbeianin-1 exhibited a rather low hemolytic activity, which was lower than 50% at 13.25 μM.

#### 4.1.3. Biosynthesis by Using Eukaryotic and Prokaryotic Expression Systems

Since the 1990s, *E. coli* has been used for recombinant expression of Cecropin and its derivatives, which effectively cleave cell membranes and combat various bacteria. Currently, the E. coli expression system has achieved the greatest number and widest distribution of antimicrobial peptides, utilizing advanced technology. Biosynthesis of antimicrobial peptides like LL37-derived MLH, Plectasin, Lactoferricin, and Buforin II has been successfully demonstrated. However, prokaryotic expression systems encounter challenges: (i) Many antimicrobial peptides exhibit broad-spectrum activity against their prokaryotic hosts; (ii) due to their positive net charge, most peptides are susceptible to degradation by endogenous proteases, a challenge mitigated by employing fusion protein strategies. Bin Dong et al. [84] through the expression system of *Escherichia coli* Rosetta synthesized ShLysG (first isolated from the hippocampus of the great belly in 2016) and subcloned its coding sequence into the pET-28a(+) plasmid to obtain the pET-28a-ShLysG vector, which was expressed and purified in *E. coli* BL21 (DE3). The results demonstrated that ShLysG, synthesized via the aforementioned method, exhibited inhibitory activity against *Listeria monocytogenes* (MIC = 20 μg/mL), *Bacillus subtilis* (MIC = 25 μg/mL), and *Staphylococcus aureus* (MIC = 40 μg/mL), among other microorganisms, and its antioxidant capacity was also well characterized, with MICs of hydroxyl group, superoxide anion, DPPH, and ABTS+ 50 values of 40, 50, 50, and 65 μg/mL, respectively. Eukaryotic expression systems, such as those using *Saccharomyces cerevisiae*, Picrytis pastoris, and C. reinhardtii, are representative examples. These systems utilize vectors such as pPICZa, pPIC9K, and pGAPZa. In comparison to prokaryotic expression systems, yeast expression systems offer advantages such as low toxicity, high activity, ease of antimicrobial peptide isolation, promotion of extracellular peptide expression, and capacity for post-translational modifications (including disulfide bond formation, O-glycosylation, and N-glycosylation). However, they are also associated with drawbacks such as slow cell growth, low yield, and the potential toxicity of certain antimicrobial peptides (e.g., beehive toxins) to the host strain [85]. Mengru Li et al. [86] successfully expressed Odorranain-C1, an α-helical cationic antimicrobial peptide derived from frog skin, using Saccharomyces cerevisiae X-33 as the expression system and pPICZα-A as the vector. The concentration of Odorranain-C1 in the expression product was approximately 14 μg/mL. The study demonstrated that recombinant Odorranain-C1 exhibits improved thermal stability compared to its natural form, as well as stability under extreme conditions including acidic, alkaline, and protease-rich environments. Moreover, Odorranain-C1 showed low hemolytic activity against hRBCs and potent inhibitory activity against a range of bacteria. Specifically, it effectively inhibited three Gram-positive bacteria (*Staphylococcus aureus*, *Bacillus subtilis*, *Listeria monocytogenes*) and three Gram-negative bacteria *(Salmonella* spp., *E. coli O157*) with MICs ranging from 8 to 12 μg/mL.

**Figure 6:**
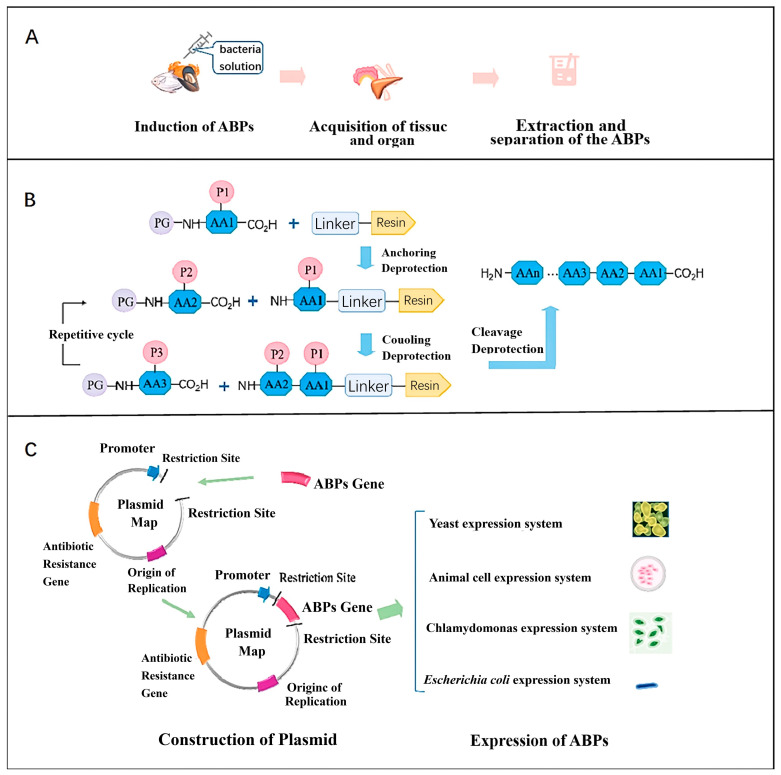
Production technologies for seafood-derived antibacterial peptides (AMPs): (**A**) biological extraction, (**B**) solid-phase synthesis (AA1: amino acid 1, PG: protecting group, P1: side-chain protecting group 1), and (**C**) recombinant protein expression [80].

Figure 6 illustrates the production techniques for these three types of AMPs.

### 4.2. Specific Applications of Typical AMPs

Antimicrobial peptides are now widely studied because they can disrupt microbial cellular structures with little effect on food. For example, *Natamycin*, an antifungal antimicrobial peptide derived from Streptomyces, has been successfully applied to the preservation of sweet potatoes. When the concentration of Natamycin in the film solution increases from 40 μg/mL to 100 μg/mL, the diameters of the zones of inhibition against *Penicillium*, *Aspergillus*, *Rhizoctonia*, and *Saccharomyces* will increase from 6.33 mm, 6.84 mm, 5.34 mm, and 5.41 mm to 13.84 mm, 13.50 mm, 14.23 mm, and 12.63 mm, respectively. At 120 days of storage, the surface mold spots of natamycin-treated sweet potatoes were much less than those of normally preserved sweet potatoes [87]. The antimicrobial and anti-biofilm potential of chia seed-derived peptides in food preservation is also significant, as reported by León Madrazo [88], and the results showed that these peptides possessed potential antimicrobial activity with minimum inhibitory concentrations ranging from 0.23 to 5.58 mg/mL, biofilm inhibition rates of more than 40%, and eradication rates of less than 20% and demonstrated that these peptides were not hemolytic or cytotoxic agents. Among other things, in microbial attack tests, KKLLKI showed good antimicrobial activity against Streptococcus enteritidis in refrigerated pork samples and Salmonella spp. in complex food matrices such as pork.

Specific studies of two of the most widely studied antimicrobial peptides (lactostreptococcal peptides and schizococcal peptides) are discussed in detail below.

#### 4.2.1. Nisin

Nisin is a polycyclic antimicrobial peptide naturally produced by certain Gram-positive bacteria, such as Streptococcus and Lactococcus. It holds the distinction of being the first antimicrobial peptide to receive GRAS (Generally Recognized As Safe) status from the FDA. Over the past four decades, nisin has been widely utilized in the food industry as a preservative. It is employed for biopreservation purposes in various food products including cheese, butter, alcoholic beverages, canned vegetables and fruits, seafood, eggs, and meat products, among others. Ana Elena Cedillo Olivos et al. [89] investigated the storage of Mexican tomato paste using different preservatives. Throughout 90 days of storage, no microbial growth was observed in samples from all storage conditions. By day 120, the control group without preservatives showed microbial presence at 2.5 CFU/mL. By day 150 at 35 °C, samples treated with potassium sorbate exhibited microbial growth at 20.0 CFU/mL, similar to samples treated with 0.1% *w/v* nisin, which also showed 20.0 CFU/mL. In contrast, the sample treated with 0.1% *w/v* nisin detected only 5 CFU/mL, while the sample with 0.2% *w/v* nisin showed no detectable microorganisms. The color, sensory evaluation, and titratable acidity results proved that Streptococcus lactis peptides were effective in food preservation. Walaa M. Elsherif et al. [90] compared the inhibitory effects of Streptococcus lactis peptides and peptide nanoparticles on *methicillin-resistant Staphylococcus aureus* (MRSA) and *E.coli O157:H7* in yogurt production and storage. Streptococcus lactis peptides inhibited MRSA at 0.0625 mg/mL, while nanoparticles had an MIC of 0.0313 mg/mL against MRSA. Streptomyces lactis nanoparticles at 0.125 mg/mL effectively deactivated MRSA in yogurt within 24 h and *E.coli O157:H7* by the 5th day. In addition, the use of pectin microencapsulation (NPM) for embedding is a good choice in order to protect lactobacillus peptides from degradation, inhibitors, and chemical reactions, as well as to increase the activity of lactobacillus peptides against foodborne microorganisms. Wenqing Liu et al. [91] utilized a pectin-based “egg carton” structure with embedded lactobacillus peptides to preserve mangoes. The experimental findings revealed that this structure, cross-linked with calcium ions (Ca^2+^), significantly reduced spoilage by 28% (*p* < 0.05) compared to the control over an 8-day storage period at 25 °C. Additionally, mangoes treated with this method maintained 6.45% soluble solid content (SSC), 2.20% titratable acidity (TA), and a hardness of 3.30 N, while limiting body weight loss to 3.40% and suppressing the increase in respiratory rate after 8 days of storage. Furthermore, the study demonstrated that this pectin microencapsulation (NPM) enhanced the activity of antioxidant enzymes and inhibited enzymes responsible for softening, suggesting potential benefits for extending mango shelf life.

#### 4.2.2. Pediocin

Pediocin, a class IIa bacteriocin with antimicrobial activity against a wide range of foodborne bacteria, is produced by Pediococcus and Lactobacillus plantarum [92] and is usually produced by Pediococcus pentosaceus and Pediococcus acidilactici to serve as a fermentation agent for inhibiting contamination during fermentation in general. Pediocin showed higher antimicrobial activity against some Gram-positive foodborne pathogens (e.g., *Listeria monocytogenes*) and Gram-negative bacteria (e.g., *Pseudomonas aeruginosa* and *Escherichia coli*) as compared to nisin [93]. Norton Komora et al. [94] combined pediocin PA-1 with high-pressure processing (HPP) and bacteriophage treatment to investigate their collective impact on fermented sausages. Over the storage period from day 30 onwards, Lactobacillaceae became the dominant bacterial family, alongside other microbiota such as Pseudomonas and Moraxella, which are associated with spoilage. By day 60, the relative abundance of bacteria in the samples was significantly lower compared to the control group (75.9% vs. 91.3%), indicating effective inhibition of spoilage bacteria species and numbers. The combination of pediocin, high-pressure processing, and bacteriophage treatment effectively delayed the spoilage of the meat products. Importantly, parameters such as color, texture, and lipid peroxidation did not show significant differences between the samples and the control group (*p* > 0.05). Taís M. Kuniyoshi et al. [92] purified pediocin PA-1 from Pseudomonas acidophilus lactis LMG 2351 and subsequently used recombinant Escherichia coli for the purification of pediocin M31L, a derivative of pediocin PA-1. They compared the anti-Listeria activity of pediocin M31-L with penocin A using a human intestinal environment model. The results demonstrated reductions in Listeria monocytogenes by 4.25 CFU/mL and 4.11 CFU/mL after 24 h, respectively. These findings indicate that pediocin M31-L exhibits significant anti-Listeria activity in the model.

#### 4.2.3. Cecropins

Cecropins are the first identified AMPs in insects known for their high efficiency, which adopt an alpha-helical conformation in membrane-like environments, efficiently disrupting bacterial membranes through permeabilization [95]. Cecropins, α-helical linear AMP (37 amino acids) lacking cysteine residues, were first isolated from *Hyalophora cecropia* moth infected with bacteria [96]. It has been reported to selectively induce apoptosis in cancer cells, but apart from clinical medicine, cecropins at very low concentrations exhibit antibacterial activity against a wide range of Gram-positive and Gram-negative bacteria [97]. Mehni Afsaneh Moslemi et al. [98] found that the causal agent of mushroom brown blotch, *Pseudomonas Tolaasii*, is susceptible to insect cecropins. They evaluated the efficacy of seven insect AMPs, including three cecropins, spinigerin, and three proline-rich peptides, for controlling P. tolaasii: In vitro assays on Petri plates showed potent inhibitory effects of cecropins against P. tolaasii with MIC values ranging from 3 to 25 µM. Other peptides presented no significant growth inhibition. Gholizadeh Abdolmajid et al. [99] cleared that cecropin B antibacterial activity on *Pseudomonas aeruginosa* was lesser than others (minimum inhibitory concentration, 0.4 μg/mL), and *Staphylococcus aureus* growth can be inhibited by Cecropin AD more than others (minimum inhibitory concentration, 0.2 μg/mL). 

## 5. Other Technical Approaches

Chemical methods for food preservation primarily involve the development and modification of organic or inorganic compounds used as additives. As society advances, there is a burgeoning interest in the development of new food additives. Chlorine dioxide, an inorganic compound known for its strong oxidizing properties, is recognized as highly efficient, low-toxicity, and rapid, making it a broad-spectrum green disinfectant. It was approved by the U.S. FDA in 1985 for disinfecting food-processing equipment and by the World Health Organization in 1992 for treating drinking water. Chlorine dioxide has since been applied widely in disinfecting drinking water, as well as in preserving fruits, vegetables, aquatic products, beverages, meat products, and antimicrobial food packaging. However, to achieve safer and more reliable food preservation, various applications of chlorine dioxide are continually being explored. Tao Wang et al. [100] enhanced the shelf life of cherry tomatoes by three days using polylactic acid microcapsules that release chlorine dioxide upon exposure to moisture. By the 18th day of storage, the decay rate of the treated tomatoes was 31.7%, which was 63.5% lower than the control group. This demonstrates the effectiveness of NaClO_2_ microcapsules in preventing fruit spoilage. Furthermore, the treated tomatoes exhibited reduced weight loss, better maintained glossy texture, and increased soluble solids content. However, chlorine dioxide, being a potent oxidizing agent, adversely affects the levels of reducing substances such as vitamin C, flavonoids, and phenols in the fruit.

In addition to photodynamics and ionizing radiation, physical methods employed for food preservation include ultrasound and high-pressure techniques. Ultrasonication induces acoustic cavitation in liquid food, where tiny bubbles form and collapse within microseconds. The effectiveness of this method depends on factors like ultrasonic intensity, process duration, temperature, food substrate, pH, and ionic strength. However, ultrasound can also disrupt protein structures and break down starches and other biopolymers. Ultra-high pressure technology involves placing vacuum-packed food into containers filled with a pressure-transmitting fluid, typically water, and subjecting them to isostatic pressures ranging from 300 to 600 MPa. This process effectively breaks non-covalent bonds, such as hydrogen bonds within enzymes, leading to their inactivation and preserving food freshness. Filipa Vinagre Marques Silva et al. [101] investigated the preservation of strawberries, apples, and pears using ultrasonication and high-pressure treatments, focusing on the inactivation of polyphenol oxidase enzymes responsible for enzymatic browning. The research revealed varying enzyme reactivity across fruits under conditions of 600 MPa and 71 °C: Pears showed 125.1% reactivity (25% activation), apples 65.8%, and strawberries 2.8%. Ultrasonication significantly reduced polyphenol oxidase activity to 3.7%, outperforming the control group at 27.8%. However, high-pressure treatment yielded mixed results, achieving 65.8% enzyme reactivity. These findings underscore the influence of fruit matrix composition on the effectiveness of preservation methods.

Biological preservation techniques are increasingly favored over physicochemical methods such as photodynamic and ionizing radiation. Plant-derived preservatives, notably essential oils, have proven effective in food preservation. The antimicrobial mechanism of essential oils has been extensively investigated, though it remains somewhat contentious. The prevailing hypothesis suggests that hydrophobic essential oils disrupt cell membranes, thereby increasing their permeability, causing leakage of cellular contents, and ultimately leading to cell death. Sara Safaeian Laein et al. [102] encapsulated Zataria multiflora Boiss essential oil (ZEO) in solid lipid nanoparticles (SLNs), significantly enhancing its antimicrobial and antioxidant activities, as evidenced by a reduction in IC50 values from 1.26 mg/mL to 0.54 mg/mL. Furthermore, ZEO-SLNs loaded with alginate were utilized to create a film that reduced bacterial counts by 1.12 log10 CFU/g compared to the control sample, effectively extending the storage duration of fresh chicken fillets.

Table 2 compares the advantages and disadvantages of these typical other antibacterial technologies.

## 6. Limitations and Disadvantages

aPDT has been under study for decades but remains primarily at the research stage in food preservation. Several factors contribute to this limitation: ① The light source’s penetration depth is restricted, resulting in uneven sterilization effects; ② aPDT is not conducive to low-oxygen environments, making it challenging to integrate with existing methods like vacuum packaging and low-temperature conditioning; ③ Exposure to light and oxygen during aPDT treatment may degrade vitamins, proteins, fats, and other nutrients in food; ④ Many natural photosensitizers extracted from plants often impart color, potentially impacting the sensory quality of food products and limiting their broader application. ⑤ Traditional small molecule photosensitizers often lack selective recognition and are prone to photobleaching, further complicating their efficacy in food preservation.

Ionizing radiation technology is internationally recognized for its sterilization abilities but is not widely used in food preservation currently due to several reasons:① Special protective measures are necessary to prevent harm to the human body when using ionizing radiation; ② The equipment and safety measures required are expensive, with costs ranging from $28 to $55 per cubic meter or $110 to $205 per ton. ③ High doses of ionizing radiation can cause significant damage to the sensory qualities, texture, and nutrients of food.

While AMPs have been studied extensively and encompass a wide variety, only nisin has been approved as a food additive. This limited adoption is primarily due to several factors: ① The specific mechanisms of action for many AMPs remain unclear; ② Some AMPs may exhibit toxic effects on host cells; ③ AMPs are susceptible to degradation by proteases; ④ Many AMPs are sensitive to salt concentration, leading to reduced activity in normal saline solutions.

## 7. Conclusions and Future Perspectives

Despite the minimal impact on food color, texture, sensory evaluation, and essential nutrients in most experiments, certain food matrices have shown slight decreases in sensory quality post-treatment, hindering the widespread adoption of new preservation technologies. Challenges persist, particularly in preserving color and texture in specific foods, limiting the applicability of emerging technologies. Further advancements are needed to enhance the universality and effectiveness of these technologies across different food types.

Nanosizing polystyrene improves its chemical performance, enhancing antibacterial efficacy. Research is crucial to refine coating and nanoparticle methods, ensuring improved stability and efficacy for broader practical use in food safety and healthcare. Nan Wang et al. [110] designed curcumin-loaded composite nanoparticles using high-hydrostatic strain-handled (HHP-treated) zein and pectin. Despite enhancements, the bioavailability of Cur remains unsatisfactory, necessitating further research into areas such as faster release and higher encapsulation rates. Nanoscience shows promise in significantly enhancing the effectiveness of photodynamic therapy in clinical medicine.

Future developments in food preservation technologies require multidisciplinary collaboration, with careful consideration of potential mutual interference between different technologies and their adverse research impacts. Understanding nanoscale interaction mechanisms should be prioritized. Promoting and applying these technologies must address cost issues, with a focus on cost reduction. Safety concerns in both food packaging and food additive forms are critical to ensure consumer health.

While there is significant homogeneity in the application research of these technologies and insufficient understanding of their mechanisms of action, future development prospects are promising. Cross-disciplinary integration across multiple fields is an inevitable trend, with research on antibacterial technologies in food preservation offering valuable insights for the medical field.

## Figures and Tables

**Table 1:** Some typical studies of natural photosensitizers for food preservation (from 1 January 2022 to 30 April 2024) [25,26,27,28,29,30,31,32,33,34,35,36,37].

PS	Matrix	Photosensitizer and Usage	Light	Cultivation Condition	Bacteriostatic Effect
Curcumin	Shrimp	8 μM Soak 20 min	455–460 nm 1.2 J/cm ^2^	Storage at 10 °C for a certain period of time	Quantity of *Vibrio parahaemolyticus* after 6 days storage < 2.0 log10 CFU/g
Curcumin	Food contact surface	15 mg/L Soak	420 nm 20 mW/cm ^2^ (5 min)	Incubate at 30 °C for 30 min	*Escherichia coli* O157 H7 CFUs reduced by 0.22 log10 CFU/mL
Curcumin + Riboflavin	Milk	250 μg/mL Solution mixing	450 nm 2.7 mW/cm ^2^ (5 min)	Incubation at 25 °C for 48 h	The average bacterial count of riboflavin is 6.95 log10 cfu/mL, while curcumin is 6.73 log10 CFU/mL
Curcumin	Sashimi salmon	500 μmol/L Soak 20 min	445–460 nm 3.80 mW/cm^2^ (60 min)	Below 4 °C, measured every two days	The counts of *Psychrophilic bacteria*, *Pseudomonas*, *Enterobacteriaceae*, and *Hydrogen sulfide bacteria* were 3.34, 2.38, 2.75, and 2.47 log10 CFU/g
Curcumin	Carrot juice	100 μM Solution mixing	455–450 nm 0.9 W/cm^2^ (30 min)	Incubated at 37 °C for 1 h	*Escherichia coli* and *Staphylococcus aureus* decreased by 2.36 log10 CFU/mL and 6.60 log10 CFU/mL
Curcumin-β-cyclodextrin	Chilled pork	Supernatant after separation of 400 mg β-cyclodextrin and curcumin solution	425 nm (45 min)	4 ± 1 °C temperature; 5565% humidity; samples are collected every two days	TVC 5.78 ± 0.17 log10 CFU/g
Curcumin	Fruit juice	10 μM Solution mixing	440 nm 3.6 × 10^−3^ W/cm^2^ (6 min)	Incubation at 37 °C for 24 h	*Staphylococcus aureus* in mango juice and pineapple juice decreased by 1.8 and 3.5 log10 CFU/mL
Chlorophyll	Fresh-cut pakchoi	1 × 10^−5^ mol/L Surface spraying	405 nm 22.27 J/cm^2^/d (12 h every day)	4 °C, Light for 12 h a day, measured every two days	The colony count decreased by 74.23%
Quercetin	Milk	75 μM Solution mixing	405 nm 80 and 120 J/cm^2^ (the former is *Escherichia coli* and the latter is *Listeria monocytogenes*)	Incubated in the dark at 37 °C for 24–48 h	*Escherichia coli* O157:H7 and *Listeria monocytogenes* decreased by 5.01 log10 CFU/mL and 1.93 log10 CFU/mL
Quercetin	White grape juice	75 μM Solution mixing	405 nm 20 and 40 J/cm^2^ (The former is *Escherichia coli* and the latter is *Listeria monocytogenes*)	Incubated in the dark at 37 °C for 24–48 h	*Escherichia coli* O157:H7 and *Listeria monocytogenes* decreased by 5.46 log10 CFU/mL and 5.98 log10 CFU/mL
Quercetin	Apple juice	50 μM Solution mixing	405 nm 19.2 mW/cm^2^; 60 J/cm^2^	Incubated at 37 °C for 24–48 h	*Escherichia coli* O157:H7 cells decreased by about 4.90 logarithms, but no cells were detected by *Listeria monocytogenes* (more than 6.73 log10 arithmic decrease)
Erythrin B	Pork	0.06 g/mL Film wrapping	400–800 nm 3.6 J/cm^2^ (30 min)	Handle for 60 min under dark and light.	Growth inhibition of bacteria 2.4 log10 CFU/m
Riboflavin	Tuna Fillet	80 μM Solution mixing	455 nm 5.2 mW/cm^2^ 30 min)	Incubate overnight at 37 °C	The maximum reduction of *salmonella cell* population was 5.02 log10 CFU/mL
Riboflavin	Fresh pork nuggets	Add an appropriate amount of riboflavin to 2% *w*/*w* chitosan solution; soak for 30 s	360 nm 15 w	Sealed in disposable Petri dishes and stored in a 4 °C refrigerator	The inhibition zones of *Escherichia coli* and *Staphylococcus aureus* coating were 6.37 ± 0.15 mm and 7.61 ± 0.32 mm
Aloe emodin	Apple juice	1 μg/mL; solution mixing	450–460 nm 40 mW/cm^2^	Incubated on solid Agar medium at 37 °C for 12 h	The survival rate of bacteria significantly decreased to about 13%

**Table 2:** Comparison of advantages and disadvantages of other typical antibacterial technologies [102,103,104,105,106,107,108,109].

Item	Advantages	Disadvantages
Chlorine dioxide	Efficient and broad-spectrum Fast and long-lasting Safety and environmental protection: It has no carcinogenic, teratogenic, or mutagenic effects on higher animal cells, sperm, and chromosomes. Non-toxic and non-irritating: It is absolutely safe in terms of acute toxicity and genetic toxicology. Wide applicability	Only at lower concentrations Complex operation process: requiring professional equipment and personnel to operate. High price Poor transportation and storage safety: prevent leaks and accidental explosions.
Combination of ultrasound and ultra-high-pressure technology	Efficiency Mildness: It causes less damage to the processed items. Environmental friendliness Safety: It is simple, radiation-free, and will not produce harmful substances. Energy saving: Cold processing methods consume less energy than heating methods, and the processing process does not produce thermal effects.	Restricted by multiple factors: such as bacterial structure, food composition, and temperature. Equipment cost: Although the cost of a single sterilization treatment is relatively low, the initial equipment investment is high.
Essential oil	Natural source: It is generally considered safer and more environmentally friendly. Wide antibacterial spectrum Low toxicity Multifunctionality: It can not only be used for food preservation but also has multiple functions such as food seasoning and processing.	Poor stability Concentration control: Improper concentration can lead to disinfection failure or harm. Potential allergic reactions

## Abbreviation

aPDT	antimicrobial photodynamic therapy	TAB	total asparagus bread powder
IR	ionizing radiation	ARP	actin-related protein
AMPs	antimicrobial peptides	MMb	3-methoxy-3-methyl-1-butanol
PS	polymeric substance	DPPH	2,2-diphenyl-1-picrylhydrazyl
EPS	extracellular polymeric substance	LEEB	low-energy electron beams
DNA	deoxyribo nucleic acid	NEB	nanosecond electron beams
UVA	ultraviolet radiation A	ALF	anti-lipopolysaccharide factors
UVC	ultraviolet radiation C	MGD	mytilus galloprovincialis defensin
SOC	spin-orbit coupling	HPLC	high-resolution liquid chromatography
LED	light-emitting diode	LC-MS	liquid chromatograph-mass spectrometer
ROS	reactive oxygen species	LL37	antibacterial protein LL-37
Curs	curcumins	PET-28(+)	PET-28A(+) plasmid
CS	corn starch	ABTS	2,2′-azino-bis(3-ethylbenzothiazoline-6-sulfonic acid
NBs	nano gas bubbles	hRBCs	human red blood cells
Oxy	oxygen	MRSA	methicillin-resistant staphylococcus aureus
FMN-	flavin mononucleotide	NPM	pectin microencapsulation
CDs	carbon dots	TA	titratable acidity
KGM	konjac glucomannan	HPP	high-pressure processing
MICs	minimal inhibitory concentrations	UHP	ultrahigh-pressure
SOD	superoxide dismutase	ZEO	Zataria multiflora Boiss essential oil
POD	polyphenol oxidase	SLNS	solid lipid nanoparticles
FDA	Food and Drug Administration	β-CD	β-cyclodextrin
EB	erythrosine B		
